# Trends of imported malaria in China 2010–2014: analysis of surveillance data

**DOI:** 10.1186/s12936-016-1093-0

**Published:** 2016-01-25

**Authors:** Sheng Zhou, Zhongjie Li, Chris Cotter, Canjun Zheng, Qian Zhang, Huazhong Li, Shuisen Zhou, Xiaonong Zhou, Hongjie Yu, Weizhong Yang

**Affiliations:** Key Laboratory of Surveillance and Early-warning on Infectious Disease, Division of Infectious Disease, Chinese Centre for Disease Control and Prevention, 155 Changbai Road, Changping District, Beijing, 102206 China; Global Health Group, University of California, San Francisco, San Francisco, CA USA; National Institute of Parasitic Diseases, Chinese Centre for Disease Control and Prevention, Shanghai, 200025 China

**Keywords:** Imported malaria, Surveillance, Elimination, Exported labour, China, Africa

## Abstract

**Background:**

To describe the epidemiologic profile and trends of imported malaria, and to identify the populations at risk of malaria in China during 2010–2014.

**Methods:**

This is a descriptive analysis of laboratory confirmed malaria cases during 2010–2014. Data were obtained from surveillance reports in the China Information System for Disease Control and Prevention (CISDCP). The distribution of imported malaria cases over the years was analysed with X^2^ for trend analysis test. All important demographic and epidemiologic variables of imported malaria cases were analysed.

**Results:**

Malaria incidence in general reduced greatly in China, while the proportion of *Plasmodium falciparum* increased threefold from 0.08 to 0.21 per 100,000 population during the period 2010–2014. Of a total 17,725 malaria cases reported during the study period, 11,331 (64 %) were imported malaria and included an increasing trend: 292 (6 %), 2103 (63 %), 2151 (84 %), 3881 (96 %), 2904 (97 %), respectively, (X^2^ = 2110.70, p < 0.01). The majority of malaria cases (imported and autochthonous) were adult (16,540, 93 %), male (15,643, 88 %), and farming as an occupation (11,808, 66 %). Some 3027 (94 %) of imported malaria cases had labour-related travel history during the study period; 90 % (6340/7034) of *P. falciparum* infections were imported into China from Africa, while 77 % of *Plasmodium vivax* infections (2440/3183) originated from Asia.

**Conclusions:**

Malaria elimination in China faces the challenge of imported malaria, especially imported *P. falciparum*. Malaria prevention activities should target exported labour groups given the increasing number of workers returning from overseas.

## Background

Malaria continues to be a major public health issue. The World Health Organization estimates that 198 million malaria cases and 584,000 malaria deaths occurred globally in 2014 [1]. The heaviest burden of malaria is in sub-Saharan Africa, which constitutes approximately 90 % of total estimated malaria deaths. Historically, malaria was one of the most prevalent parasitic diseases in China. Yet, after decades of control efforts, the malaria burden has been dramatically reduced from a peak malaria incidence of 2961/100,000 population in 1970 to 0.1/100,000 in 2014 [2–4]. The total population at risk in China is shrinking and since 2010 only Yunnan province reports autochthonous *Plasmodium falciparum* malaria. As a result of this progress, starting in 2010, the Chinese Government began implementing a malaria elimination plan to achieve malaria-free status by 2020 [5].

*Plasmodium vivax* has historically been the predominant *Plasmodium* species in China [2, 3]. The percentage of *P. vivax* varied from 80 to 95 % of total malaria cases annually with the remaining percentage being *P. falciparum*. However, with a decrease in autochthonous malaria cases, the situation in China is changing and total falciparum malaria is increasing. Imported *P. falciparum* malaria cases have been reported recently in all provinces in China [6–8], which poses a major challenge due to its potential for fatal cases [9].

Another issue is the risk of malaria re-introduction due to importation, which has been identified in China and elsewhere, for example in Greece [10, 11]. The re-emergence of imported malaria in central China worsened between 2000 and 2009 as the total malaria incidence rate reached a peak of 5.9/100,000 population up from 1/100,000 [12, 13]. Thus far the risk of imported malaria from other endemic settings continued to increase and this is a major challenge for malaria elimination in China. The objectives of the current study were to better understand and address this challenge, the epidemiological profile and trends of imported malaria in China from 2010 to 2014.

## Methods

### Surveillance data

Malaria has been a notifiable disease in China since 1956. Currently, over 68,000 health facilities report notifiable diseases to the national, real-time, internet-based disease reporting system, known as the China Information System for Disease Control and Prevention (CISDCP). One-hundred percent of the Centres for Disease Control and Prevention (CDC), 98 % of hospitals above county level and 94 % of hospitals below county level report to the CISDCP [14]. According to the Technical Scheme of Malaria Elimination in China [15], all positive blood smears and 10 % of negative blood smears are checked by county CDC for quality assurance. The provincial CDC rechecked blood smears of all possible, probable and confirmed cases reported from counties with a malaria incidence rate lower than 1/10,000 population annually over the past 3 years. A retrospective analysis using routine surveillance data from the CISDCP during the period of 2010–2014 was carried out. The basic malaria case information included: date of birth, gender, occupation, usual residence, details of malaria illness such as date of onset of symptoms, date of diagnosis, date of treatment, and method of diagnosis.

### Epidemiological data

Additional epidemiological information was obtained from the Information System for Parasitic Disease Control and Prevention (ISPDCP), which is entered by the local county-level CDC after interviewing malaria patients once the county hospital submits a malaria case report to the national CISDCP system. A national case investigation form is used to collect additional epidemiological information, including details on travel history, such as date of departure from China, date of arrival back to China, countries visited, and purposes of travel. Based on this information, county-level CDC staff can determine if the malaria case is autochthonous or imported. For those with complete details, the epidemiological information is matched to surveillance data by a unique case reporting code. For those without complete case details, the data are matched by name, usual residence and household address.

### Malaria case definition

Malaria cases with laboratory-confirmed *Plasmodium* infections notified to the CISDCP during 2010–2014 were included in the study. Laboratory confirmation denotes parasites in microscopic examination of a blood smear. Cases were classified as Chinese or foreigner. According to the Technical Scheme of Malaria Elimination [15], an imported case was defined as a malaria infection traced to an origin in a malaria-endemic area outside China and within 1 month after returning from the endemic area. An autochthonous case is defined as a malaria infection from local transmission with no history of travel and locally acquired transmission cannot be disproven.

### Inclusion and exclusion criteria

All patients confirmed by microscopy and audited by the CISDCP from the period of January 2010 through December 2014 were included in this study. Probable and possible cases in the CISDCP were excluded from the study. Duplicate cases were excluded by identifying each condition of unique case reporting code, personal identification code, and patient’s name plus current residence. Due to poor supporting information, relapse and recurrence cases were also excluded.

### Statistical analysis

Descriptive statistics was done for all important variables. Year-on-year change was done for the distribution of imported malaria as well as falciparum malaria over the study period. The trends of this distribution was assessed with X^2^ for trend analysis test. Statistical significance was set at 5 % level, unless otherwise stated. Data were analysed with Stata version 10.0 (Stata Corporation, College Station, TX, USA).

## Results

### General surveillance

**Fig. 1:**
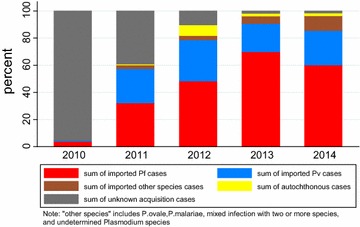
Acquisition of malaria cases in China by species (2010–2014)

Between 2010 and 2014, 17,725 malaria cases were diagnosed by microscopy; 17,079 (96 %) cases were Chinese and 646 (4 %) were foreign nationals; 11,331 (64 %) imported malaria cases were reported with an increasing trend from 2010 to 2014 compared to the total: 292 (6 %), 2103 (63 %), 2151 (84 %), 3881 (96 %), 2904 (97 %), respectively, (X^2^ = 2110.70, p < 0.01) (Fig. 1). A total of 362 (2 %) autochthonous malaria cases were reported from 2011 to 2014 compared to the total: 18 (0.5 %), 199 (8 %), 83 (2 %), 62 (2 %), respectively, (X^2^ = 8.76, p < 0.01); 6032 (34 %) of reported malaria cases were without a known source of infection from 2010 to 2014: 4474 (94 %), 1233 (37 %), 218 (8 %), 64 (2 %), 43 (1 %), respectively, (X^2^ = 9456.50, p < 0.01). Total malaria incidence (per 100,000 population) in China had a general decreasing trend from 2010 to 2014: 0.55, 0.30, 0.18, 0.28, 0.21 respectively. A total of 98 deaths occurred during the period of 2010–2014: 15, 27, 15, 20, 21 respectively, (X^2^ = 1887.74, p < 0.01).

### Characteristics of demographic and epidemiological distribution

**Fig. 2:**
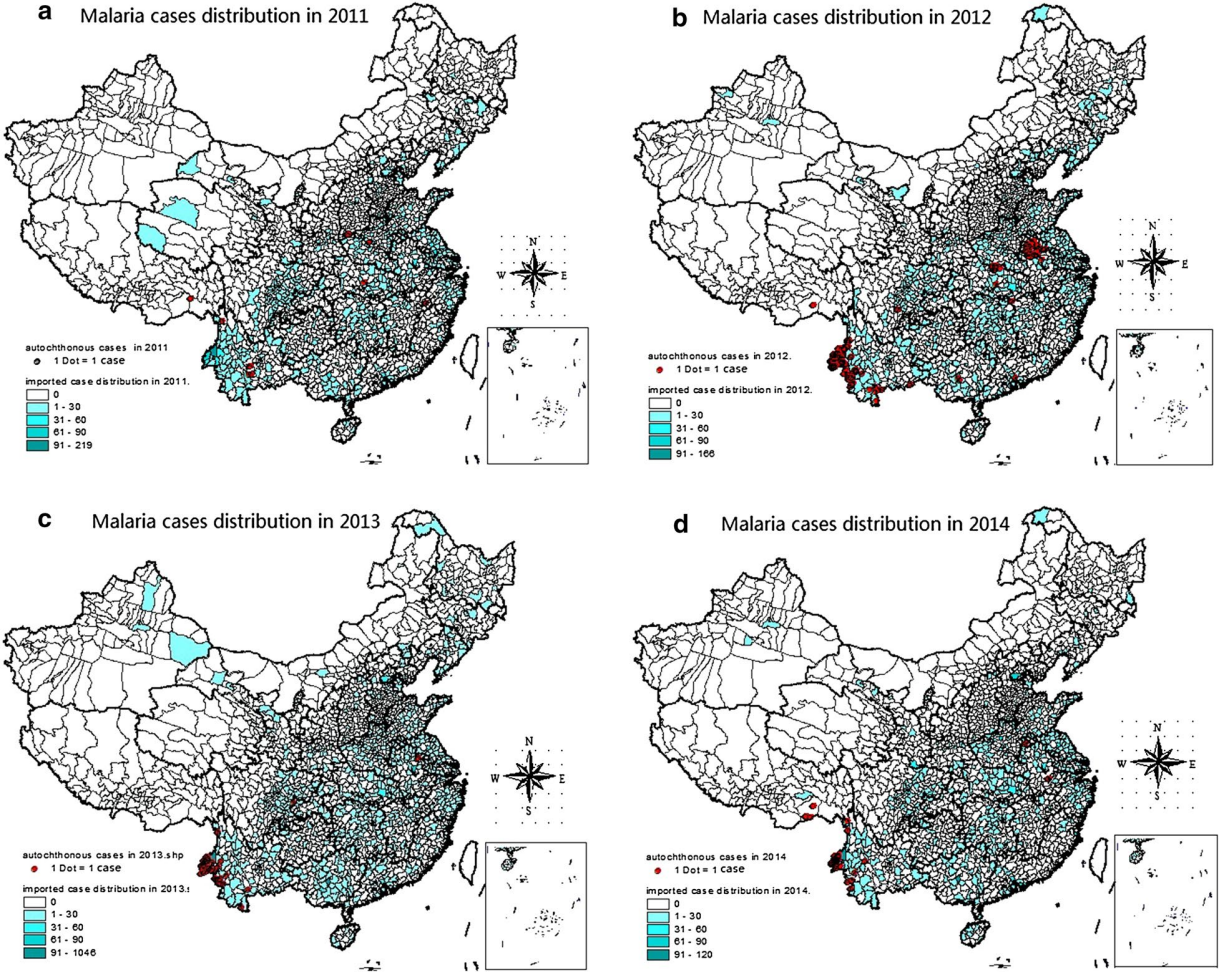
Distribution of imported malaria in China by county, 2011–2014

Between 2010 and 2014, altogether 17,725 malaria cases were reported. The median age of these cases was 37.2 years (range 37.0–37.4) and the majority was males (15,643, 88 %) and farmers by occupation (66 %, 11,808) (Table 1). Sixty-nine percent (12,253) of total autochthonous and imported malaria cases were reported in Anhui, Guangxi, Henan, Jiangsu, Sichuan, and Yunnan provinces. Total imported malaria cases were reported in 1499 (51 %) counties from all 31 provinces in China (Fig. 2).

**Table 1:** Demographic characteristics of malaria cases in China, 2010–2014

Variable	Categories	2010		2011		2012		2013		2014	
		No.	%	No.	%	No.	%	No.	%	No.	%
Sex (N = 17,725)	Male	3704	77.72	2873	85.66	2363	92.02	3847	95.51	2856	94.92
	Female	1062	22.28	481	14.34	205	7.98	181	4.49	153	5.08
Age (N = 17,725)	<10	237	4.97	117	3.40	36	1.40	22	0.55	16	0.53
	10–19	365	7.66	147	4.38	106	4.13	92	2.28	47	1.56
	20–29	1014	21.28	799	23.82	593	23.09	928	23.04	656	21.80
	30–39	1121	23.52	870	25.94	738	28.74	1159	28.77	802	26.65
	40–49	1052	22.07	967	28.83	797	31.04	1415	35.13	1071	35.59
	50–59	464	9.74	248	7.39	243	9.46	378	9.38	372	12.36
	≥60	513	10.76	206	6.14	55	2.14	34	0.84	45	1.50
Occupation (N = 17,725)	Farmer	2323	48.74	1643	48.99	1098	42.76	2207	54.79	1260	41.87
	Labourer	1107	23.22	569	16.96	392	15.26	588	14.60	405	13.46
	Worker	326	6.84	292	8.71	325	12.66	419	10.40	433	14.39
	Officer	86	1.80	100	2.98	110	4.28	138	3.43	113	3.76
	Other	924	19.39	750	22.36	643	25.03	676	16.78	798	26.52
Purpose of travelling overseas (n = 3220)	Labour export	29	74.36	347	91.80	446	91.21	1525	96.15	680	93.41
	Business trip	1	2.56	9	2.38	7	1.43	18	1.13	11	1.51
	Tourism	1	2.56	4	1.06	5	1.02	10	0.63	7	0.96
	Study	4	10.26	2	0.53	9	1.84	8	0.50	0	0
	Other	4	10.26	16	4.23	22	4.50	25	1.58	30	4.12
Days staying overseas (n = 2124)	Days										
	≤30	6	6.59	17	11.72	18	7.17	30	2.36	27	7.34
	31–180	28	30.77	44	30.34	76	30.28	207	16.31	113	30.71
	181–365	25	27.47	40	27.59	69	27.49	516	40.66	104	28.26
	≥366	32	35.16	44	30.34	88	35.06	516	40.66	124	33.70

### *Plasmodium* species

Of 17,278 cases with identified parasite species (97 % of total confirmed cases), *P. falciparum* accounted for 49 % (8631) and *P. falciparum* incidence (per 100,000 population) between 2010 and 2014 was 0.08, 0.10, 0.10, 0.21, 0.13 respectively. The share of *P. falciparum* reached a high of 71 % of the total in 2013, up from 24 % in 2010. Among 11,331 total imported malaria cases from 2010 to 2014, *P. falciparum* was the highest at 7218 cases (64 %).

*Plasmodium vivax* accounted for 45 % (7963) and the total share of *P. vivax* declined from 69 to 28 % from 2010 to 2014. Among 11,331 total imported malaria cases from 2010 to 2014, *P. vivax* cases were 3346 (30 %).

*Plasmodium ovale* and *Plasmodium malariae* accounted for 2.7 % (512) and all *P. ovale* and *P. malariae* cases were imported from 2010 to 2014. The percentage of *P. ovale* increased from 0 to 7 %, and the percentage of *P. malariae* increased from 0.02 to 2 % from 2010 to 2014. Mixed infections with two or more species accounted for 1 % (171) and the majority (156) were imported cases. One imported case of *Plasmodium knowlesi* was identified, and 447 (3 %) malaria case species were unidentified.

### Characteristics of travel-related information

In those 3220 (18 %) cases with known reasons for travelling, the vast majority (3027, 94 %) was exported labour (i.e., working overseas) (Table 1). Among the 2124 cases (12 %) with available information on staying overseas: 98 (4.6 %) cases stayed less than 30 days, 468 (22.0 %) stayed less than 180 days, 754 (35.5 %) stayed less than 365 days, 804 (37.8 %) stayed more than 365 days.

Study results show that of the 4310 imported malaria cases where complete patient information was captured, 321 (7 %) had malaria symptoms before their arrival in China after staying overseas, and 3378 (78 %) had symptoms within 30 days after their arrival from staying overseas; 3004 (84 %) of imported *P. falciparum* cases and 227 (50 %) of imported *P. vivax* cases had malaria symptoms within 1 month of their arrival from staying overseas (Table 2).

**Table 2:** Interval days from date of arrival to date of symptom onset in China during 2010–2014

Interval (days) (n = 4310)	*P. falciparum*	%	*P. vivax*	%	Other *Plasmodium* ^a^	%	Total	%
≤−1	266	7.49	43	9.58	12	3.86	321	7.45
0	263	7.41	14	3.12	15	4.82	292	6.77
1–30	2741	77.21	213	47.44	132	42.44	3086	71.60
31–60	195	5.49	70	15.59	50	16.08	315	7.31
61–90	43	1.21	36	8.02	21	6.75	100	2.32
≥91	42	1.19	73	16.26	81	26.04	196	4.55

^a^Others including *P. ovale*, *P. malariae*, mixed infection with two or more species, and undetermined *Plasmodium* species

### Region of infection acquisition

Of the 11,331 imported malaria cases, 96 % (10,925) had country information available on the location of suspected infection acquisition. Between 2010 and 2014, imported malaria case infections were acquired from 74 countries. Acquired infections were the highest among those travelling to Africa: 7679 (70 %), second highest were from Asian countries with 3208 (29 %) cases, 28 (0.26 %) were from Oceanic countries and ten (0.09 %) from South America. Malaria cases imported from Angola, Equatorial Guinea, Ghana, Myanmar, and Nigeria accounted for 66 % (7255) of total imported cases from 2010 to 2014. Ninety percent (6340/7034) of falciparum malaria cases were acquired in Africa, whereas 77 % of vivax (2440/3183) malaria cases originated from Asia (Table 3).

**Table 3:** Imported malaria country of acquisition in China during 2010–2014 by *Plasmodium* species

Country of acquisition (n = 10,925)	*P. falciparum*	%	*P. vivax*	%	Other *Plasmodium* ^a^	%	Total	%
Africa								
Ghana	1547	21.99	123	3.86	112	15.82	1782	16.31
Angola	932	13.25	51	1.60	80	11.30	1063	9.73
Equatorial Guinea	789	11.22	61	1.92	111	15.68	961	8.80
Nigeria	800	11.37	60	1.89	76	10.73	936	8.57
Cameroon	237	3.37	15	0.47	27	3.81	279	2.55
Congo	197	2.80	12	0.38	22	3.11	231	2.11
Liberia	168	2.39	25	0.79	26	3.67	219	2.00
Ethiopia	39	0.55	167	5.25	7	1.00	213	1.95
Guinea	136	1.93	9	0.28	15	2.12	160	1.46
Mozambique	123	1.75	9	0.28	9	1.27	141	1.29
Sierra Leone	121	1.72	9	0.28	11	1.55	141	1.29
CongoDR	109	1.55	13	0.41	13	1.84	135	1.24
Other African countries	1142	16.24	166	5.22	110	15.54	1418	12.98
Asia								
Myanmar	571	7.84	1893	59.47	49	6.92	2513	23.00
Indonesia	57	0.80	177	5.56	25	3.53	259	2.37
Cambodia	14	0.20	144	4.52	5	0.71	163	1.49
Laos PDR	14	0.19	86	2.70	1	0.14	101	0.92
Pakistan	5	0.07	67	2.10	3	0.42	75	0.69
India	5	0.07	47	1.48	1	0.14	53	0.49
Other Asian countries	14	0.20	26	0.82	4	0.56	44	0.40
Oceania	10	0.14	18	0.57	0	0	28	0.26
South America	4	0.06	5	0.16	1	0.14	10	0.09

^a^Others including *P. ovale*, *P. malariae*, mixed infection with two or more species, and undetermined *Plasmodium* species

## Discussion

Malaria incidence in China has been decreasing since 2010, and this study presented results on a changing malaria situation in China. Historically, *P. vivax* has been the predominant *Plasmodium* species in China, varying from 77 to 94 % annually between 2001 and 2009. The relative proportions of falciparum and vivax malaria cases have reversed between 2010 and 2014, and currently *P. falciparum* is the predominant species of all malaria cases (autochthonous and imported) in China. The changing proportion of *Plasmodium* malaria has been mainly due to effective malaria control activities nationwide. Moreover, the total proportion of *P. ovale* and *P. malariae* cases increased during this period. As of 2014, the only remaining autochthonous malaria cases were identified in Yunnan and Tibet provinces, with Yunnan being the last stronghold for ongoing *P. falciparum* malaria transmission.

Imported malaria cases had different characteristics compared to autochthonous malaria cases. Autochthonous malaria cases were typically identified among all age groups, originated in specific endemic areas, and during clear seasonal peaks. Major characteristics of imported cases included being adult, male, and farming as an occupation with reported labour-related travel history to other countries. Additional characteristics of imported cases included being reported in the non-transmission season, and were distributed nationwide, even in the areas with no history of malaria transmission [16–19]. This is a challenge for passive surveillance systems in those counties or provinces currently without malaria transmission because of their poor capacity of case detection as total malaria incidence reduces to zero in many counties. Maintenance of case detection in certain health facilities should be a critical activity where surveillance was taken as one intervention during malaria elimination in China.

Between 2010 and 2014, imported *P. falciparum* was reported in all provinces in China, largely due to importation from other countries, especially from Africa where *P. falciparum* is the main parasite. This may be partly attributed to an increasing number of Chinese labourers who visit Africa given the recent cooperation between Africa and China. Tatem et al. indicated that certain countries in West African and Central Asia are more strongly connected by high levels of infection movement than others [20]. This study found that 94 % of imported cases had recent labour-related travel history. Similarly, Liu et al. indicated that imported malaria cases in Jiangsu Province, China were mostly related to the export of labour [21]. Statistics from the China Ministry of Commerce shows that legal export of labour overseas included approximately 800,000 individuals annually over the past 5 years and the number of export labourers continues to rise each year [22]. Given this trend, international population movement between China and other malaria-endemic countries will be become even more frequent. The study findings presented the diversity of malaria importation from both African and Asian countries. Ninety percent of imported falciparum malaria came from African countries thereby increasing the chance of potentially fatal cases [9]. Intervention regarding malaria among this internationally moving population should be undertaken. Malaria intervention in the border areas should be strengthened.

Compared to those travelling for tourism or business, overseas labourers stayed for longer periods, which possibly contributed to an increased risk of contracting malaria. Study findings support the targeting of malaria prevention activities in these higher risk groups. To reduce the burden of imported malaria cases in Chinese workers coming from other countries after long stays, interventions should be specific Chinese export labourers. Within this high burden group, evidence has shown there is poor knowledge and awareness of malaria prevention methods [23, 24]. Chemoprophylaxis alone may not be a good choice due to poor understanding of benefit of taking prophylaxis and the potential negative side effects, such as resistance to anti-malarial drugs. Given the increase in numbers of overseas labourers, the supply of chemoprophylaxis in these groups would also be a challenge. Chinese export labourers work mostly in construction and mining activities, therefore, occupation-based vector control methods should be considered [25, 26], including specific behaviour change communication activities.

Furthermore, the seasonal distribution of imported malaria into China fluctuated very little. However, within four to 5 weeks, 874 imported malaria cases in 2013 were detected after 4052 Chinese gold miners returned to China after working in Ghana [27]. This event indicated that timely detection and removal of new infections is critical to prevent re-establishment of transmission [28].

This study’s findings show that 15 % of imported *P. falciparum* cases and 60 % of imported *P. vivax* cases had symptoms more than 30 days after returning from abroad. On the one hand, it indicated the definition of imported malaria should be revised. During the malaria control phase, travel history within the previous month is an appropriate definition to determine imported malaria. However, in the malaria elimination phase, the definition of imported malaria must include up to 3 months of travel history. On the other hand, it also indicated those cases should be identified as early as possible by active case detection, including reactive case detection and proactive case detection [26].

To conduct effective surveillance in this malaria elimination setting, new strategies and activities are needed. Although total malaria incidence is decreasing in China, highly sensitive diagnostics (such as PCR/LAMP) that are field-friendly, as well as genotyping techniques, are needed to identify asymptomatic and sub-patent infections and to distinguish the origin of those infections [29]. With improved rapid reporting capacity and targeted active and passive surveillance activities, responding to imported cases should be greatly improved among the target population at risk after returning from malaria-endemic countries [30, 31].

Study limitations should be mentioned. National surveillance data in China are routinely collected using standardized forms. However, some missing epidemiological data were identified in ISPDCP due to a loss-to-follow-up after treatment in the health facility. Since ISPDCP was established in late 2011, malaria case investigation data collected prior to this starting were subsequently added to the ISPDCP database. Therefore, data in 2010 and 2011 may not be a reliable source of autochthonous cases, especially in some geographic areas of China. For example, the rate of missing case classification of case investigation was 17 % from 2011 to 2014. However, the ISPDCP database is the best source for national level data in China and there is no evidence to suggest that malaria cases with missing travel history information were systematically different than those with complete reports. Further, this study presented data showing that the quality of the malaria case information captured in the surveillance system has improved over the study period. Both imported and autochthonous malaria cases may be underestimated in 2010 and 2011 due to misclassification bias. In those provinces that have eliminated falciparum malaria, the identification of the parasite may be easier once travel history to endemic areas is considered. However, China’s border areas with Laos, Myanmar and Vietnam make it more difficult to ascertain imported malaria. For example, 74 % of these cases reported in 2010 had an unknown origin of infection. Since China began to pursue malaria elimination in 2010, improvements to the epidemiological survey form have been made and information collection improved. New factors were included in the revised case investigation forms, namely the origin of infection. The missing rate of origin of infection improved since 2011 and was only 1 % of the total in 2014. Another limitation was the result from current definition of case classification in China: 23 % of total imported cases with missing infection locations were partly due to the definition.

## Conclusions

Since the Action Plan for Malaria Elimination in China (2010–2020) was issued in 2010, malaria epidemiology in China has changed dramatically. In Yunnan and Tibet provinces, where both imported and autochthonous malaria exists, cross-border movement and malaria importation will continue to pose a challenge. A continuous influx of imported malaria infections increases the potential for malaria re-introduction to all receptive areas of China. For China to reach its goal of malaria elimination, it must target those populations that travel abroad for economic reasons with effective malaria prevention strategies.
